# Integrated Care’s New Protagonist: The Expanding Role of Digital Health

**DOI:** 10.5334/ijic.6437

**Published:** 2021-10-13

**Authors:** Carolyn Steele Gray

**Affiliations:** 1 Bridgepoint Collaboratory for Research and Innovation, Lunenfeld-Tanenbaum Research Institute, Sinai Health System, US; 2 Institute for Health Policy, Management and Evaluation, Dalla Lana School of Public Health, University of Toronto, CA

**Keywords:** digital health, implementation, integrated care, system transformation

## Abstract

Digital health technologies hold significant promise to advance both functional and normative health and social care integration. The COVID-19 pandemic has created a window of opportunity to rapidly advance the adoption of digital solutions which can improve activities that support integration at clinical, professional, organizational and system levels. Global examples demonstrate how the pandemic has also created opportunities to use technology to address core values of integrated care like person-centredness and coordination. However, rapid and reactive changes could lead to increased fragmentation and exacerbate health inequity. This perspective paper outlines some of the opportunities and threats to advancing integrated care presented by the rapid adoption of digital health tools, suggesting we maintain a long view to ensure the stage we set today will mean greater integration tomorrow.

## Introduction

In September of 2019, the first webinar of the International Foundation of Integrated Care Special Interest Group on Digital Health Enabling Integrated Care proposed a definition to provide clarity on the role of technology in these models. It was suggested that digital health enabling integrated care can be defined as

*the use of digital health technologies to enable and support the functional activities and processes, as well as normative values and culture put in place to achieve the aims of an integrated model of care*.

This proposed definition builds on two foundational ideas. First, that technologies need to align to activities or tasks of users and organizations to add value [8], as suggested by the Task-Technology Fit model presented by Goodhue and Thompson in 1995 [9]. Second, that the notion of “value” extends beyond discrete actions or processes, and includes attention to the moral values, beliefs, and norms that are interwoven into a model of care at individual, organizational, and societal levels. These dual roles for digital health solutions align with Valentijn’s Rainbow Model of Integrated Care which suggests both functional and normative mechanisms are required for integration to occur [13]; and further builds on Goodwin’s argument that digital health can act as both the “grease” (functional) and “glue” (normative) of integrated care systems [14].

By supporting functional and normative changes digital health solutions can act as a crucial factor in health system transformation. Large international comparative studies of models of integrated care have found implementation of digital health to be oftentimes underwhelming, owing to challenges in funding, system limitations, spotty interoperability and infrastructure, limited training or support, and deficient policies to enable these transformations [15,16]. In the European Commission’s 2018 Integrated Care Assessment [16], only 4 of 12 cases were rated as having a high maturity in the use of information and digital health tools. Furthermore, underutilization of information technology and lack of interoperability are identified as the main organizational barriers to advancing integrated care across the 30 countries studied.

A deep dive into cases of integrated care in Canada and New Zealand sought to understand how digital health tools were being used to advance 9 models of integrated care [17]. These models were studied as part of the iCOACH project [18], and were selected for their exemplary work in integrated community-based primary health care delivery [19]. While there were some innovative uses of digital health solutions in these cases, generally technologies were being used to support old (previously siloed) ways of working, impeding their ability to advance a more mature integrated care model. Similar to the European studies, barriers such as lack of interoperability, enabling policies, and support needed to improve engagement stood in the way of more advanced adoption.

## Enter COVID: Digital Health’s big break

The global shock of COVID-19 hurled digital health into centre stage in a time of lockdowns and quarantines. After two decades of snail’s pace progress [20], there was an explosion of digital health adoption and implementation. For example virtual care use in ambulatory settings in Ontario, Canada increased from 1.6% of visits prior to COVID-19 to 70.6% in the first quarter of 2020 [21]; a trend seen globally [22,23]. But the question remains ***how will this shift impact advancement of integrated care?*** The remainder of this perspective outlines opportunities and cautions for the road ahead; pulling from the emergent growing literature regarding digital health use since the advent of COVID-19 to discuss how new trends may advance or hinder the functional and normative mechanisms that underpin integrated care.

## Opportunities in a rapid, adaptive, and increasingly open system

Healthcare systems, organizations, and providers worldwide have demonstrated an exceptional ability to rapidly change and adapt processes and activities to meet the needs of local communities during the COVID-19 pandemic. Many of these shifts have opened opportunities for digital health tools to activate functional and normative mechanisms of integrated care. ***Box 1*** outlines several functional activities enabled by digital health since the start of COVID-19.

Box 1 Digital health tools enabling activities of integrated care since the onset of COVID-19.

– *Clinical level*: An unprecedented increase in the use of virtual care across sectors [1–4] and the use of patient portals to access information [5].
– *Professional level*: Providers are using more tools to enable collaboration and teamwork through technology enabled referral, consultation, and care plan meetings/rounds [3,6].
– *Organizational level*: Health information data sets are being integrated to meet public health reporting needs [7], however infrastructure barriers to interoperability remain a significant barrier [4,10].
– *System level*: A rapid change in policies to enable digital health use, including changes to funding models to support wider adoption [11,12].

From a normative standpoint, regions, health, and social care organizations, and those delivering services had to adopt an adaptive mindset to be able to rapidly meet evolving needs of their communities [24]. One integrated model in the east end of Toronto, Canada’s largest city, was able to quickly adapt; leveraging existing integration activities to mount a rapid response to the pandemic. Building on existing partnerships and resources this model was able to “vastly expand care options” to homebound patients through use of virtual care and teams, modify care spaces to better support digital delivery while addressing social distancing requirements, and use connected data systems to inform decision-making aligned to a learning health system approach. These shifts were iterated and tailored to the needs of the community and are expected to help accelerate integration over the longer term [25]. This type of adaptive mindset has been argued to be crucial to advance transformations in complex interventions and systems like health and social care [26].

Another important normative shift has been greater attention to holistic person-centred and compassionate care delivery [2,6,27], and an encouraging rise in acknowledgment of health equity as many systemically marginalized populations were disproportionately affected by the pandemic [21,25]. New programs to address person-centredness and equity have been emerging worldwide. For example, to address the inequitable impact of COVID-19 the University Health Network in Toronto, Canada established the PHONE-CONNECT program, which distributes donated cell phones to vulnerable patients discharged from the emergency department [28]. Internationally many countries have begun to take measures to address health equity through expanding health coverage, improved coordination of services, and increasing social supports [29]. Examples like these demonstrate how values of equity, person-centredness and collaboration are becoming embedded into health care delivery; values which have been identified as drivers of integrated models of care [30].

### Threats to coordination, equity, and the role of digital health tools

Many of the same opportunities for digital health to advance functional and normative mechanisms that drive integration can also become threats to a longer-term vision. First, while digital solutions can support greater coordination, many jurisdictions still face a lack of infrastructure for sharing health data between organizations which can lead to poor handoffs, inefficiency, and ultimately further fragmentation [21]. Second, rapid adoption can lead to a reductionist view of technology, seeing it as just a tool to enable information sharing and communication. While this can support functional mechanisms of integration, it misses the important point that technology is a social artefact which can mirror and shape norms and values of the systems and people in which it is placed [31,32].

Finally, both these challenges can exacerbate health inequity at the individual level due to unequal access to technologies and disparate digital literacy [2,21,33,34]. From a population health perspective, using available data to quickly drive decision-making may not sufficiently attend to inherent biases that may exist within that data [35]; potentially further entrenching inequity in the system. Despite advances to address health and digital health equity noted above, these challenges persist and will require consistent attention and iteration of possible solutions. Shaw and colleagues offer three strategies that can help promote health equity in the context of virtual care which can be adopted even in a rapid change environment [36]:

1. Make interfaces and workflows simply and easy to follow
2. Bring on digital “liaisons” to assist newer users as they adapt to new tools
3. Have marginalized communities drive implementation through engagement and evaluation

## What’s next? Setting the stage now for improved integrated delivery tomorrow

Path dependency theory, grounded in a historical institutionalism view, encourages us to recognize that the choices we make today set the stage for the story we will tell tomorrow [37]. Simply put, how we implement digital health tools can have long-term implications for our systems, creating either opportunities for growth, or blinders on what we see as possible. Now more than a year since the onset of COVID-19 the pace of change is beginning to slow, yet there remains an opportunity to advance on what has been learned and capitalize on this window of opportunity afforded to the implementation of digital health solutions. Asking questions today about how technologies that are developed and implemented advance the functional activities and align to the core values of integrated models of care is one way to ensure that digital health will play its part towards greater integration of health and social care services.
